# Prediction of Sickness Absenteeism, Disability Pension and Sickness Presenteeism Among Employees with Back Pain

**DOI:** 10.1007/s10926-013-9454-9

**Published:** 2013-06-18

**Authors:** Gunnar Bergström, Jan Hagberg, Hillevi Busch, Irene Jensen, Christina Björklund

**Affiliations:** Division of Implementation and Intervention Research, The Institute of Environmental Medicine, Karolinska Institutet, PO Box 210, 171 77 Stockholm, Sweden

**Keywords:** Back pain, Prognosis, Yellow flags, Occupational setting, Sickness absenteeism, Sickness presenteeism

## Abstract

*Purpose* The primary aim of this study was to evaluate the predictive ability of the Örebro Musculoskeletal Pain Screening Questionnaire (ÖMPSQ) concerning long-term sick leave, sickness presenteeism and disability pension during a follow-up period of 2 years. *Methods* The study group consisted of 195 employees visiting the occupational health service (OHS) due to back pain. *Results* Using receiver operating characteristic (ROC) curves, the area under the curve (AUC) varied from 0.67 to 0.93, which was from less accurate for sickness presenteeism to highly accurate for the prediction of disability pension. For registered sick leave during 6 months following the baseline the AUC from the ROC analyses was moderately accurate (0.81) and a cut off score of 90 rendered a high sensitivity of 0.89 but a low specificity of 0.46 whereas a cut off score of 105 improves the specificity substantially but at the cost of some sensitivity. The predictive ability appears to decrease with time. Several workplace factors beyond those included in the ÖMPSQ were considered but only social support at the workplace was significantly related to future long-term sick leave besides the total score of the ÖMPSQ. *Conclusions* The results of this study extend and confirm the findings of earlier research on the ÖMPSQ. Assessment of psychosocial risk factors among employees seeking help for back pain at the OHS could be helpful in the prevention of work disabling problems.

**Electronic supplementary material:**

The online version of this article (doi:10.1007/s10926-013-9454-9) contains supplementary material, which is available to authorized users.

## Introduction

Psychosocial factors have been shown to have an impact on the course of acute/subacute neck-/low-back pain, NP/LBP [1–3]. The Örebro Musculoskeletal Pain Screening Questionnaire (ÖMPSQ), is an instrument developed for the early detection of individuals with increased risk of a poor prognosis on variables such as pain, disability and sickness absenteeism [4]. Several studies have been reported on the utility and predictive validity of the ÖMPSQ among primary care patients with acute or subacute pain [5, 6]. In a recent systematic review the pooled sensitivity of the ÖMPSQ was 0.59 (CI 0.43; 0.74) and the pooled specificity was 0.77 (0.66; 0.86) [6]. To the best of the authors’ knowledge, no studies have been carried out among employees seeking help for their NP/LBP at the occupational health service (OHS). The OHS, with its knowledge about, and access to, the employee’s workplace could offer an excellent starting point for preventive efforts in the work environment.

The ability of different versions of the ÖMPSQ to predict self-reported sickness absenteeism has been presented in earlier research [4, 7–13]. Even though self-reported sick leave has been shown to correlate acceptably well with register data [14, 15] it may be a methodological strength also to evaluate the ÖMPSQ against register data and, additionally, to include information on its ability to predict disability pension which has not been evaluated in previous studies. Furthermore, with one exception [10] the follow-up periods in earlier studies have been 1 year or shorter and no studies of the ÖMPSQ have gathered information on sickness absenteeism for two consecutive years.

During recent years there has been a growing interest among researchers in sickness presenteeism, that is, going to work despite illness [16, 17]. For the individual a decrease in sickness absenteeism may not necessarily indicate better health or more successful coping strategies. Conversely, the employee may keep on going to work due to individual or organizational factors such as job insecurity, financial problems or having to catch up on work after a period of absence [18]. Under such circumstances sickness presenteeism may actually be a better indicator of health than sickness absence [19, 20]. Because of this, sickness absenteeism and sickness presenteeism can be seen as complementary aspects of an individual’s perceived functioning (or limitations) at work.

Sickness presenteeism has been studied from an epidemiologic perspective concerning its determinants and prevalence (mostly represented by European researchers) and from an economic perspective related to its impact on the productivity of an organization (mostly carried out by North American researchers) [17, 21]. In this study we conceptualize sickness presenteeism as going to work despite being so ill that the employee considers that he/she should have taken sick leave. However, other definitions exist which is more related to the mentioned economic perspective of sickness presenteeism and include health limitations that may not be considered as “enough” to justify sickness absenteeism but nevertheless affects productivity.

The primary aim of this study was to evaluate the predictive ability of the ÖMPSQ concerning long-term sick leave, sickness presenteeism and disability pension during a follow-up time of 2 years. The study group consisted of employees seeking help due to NP/LBP at their OHS. We also purported to evaluate whether the predictive ability of the ÖMPSQ concerning sick leave was affected when adjusting for some potential predictors of sick leave in the work environment [22, 23].

## Methods

This prospective study is based on material gathered during a study titled “Work and Health in the Processing and Engineering Industries, the AHA study” [24]. This study was carried out at four workplaces within the private industry sector; two were paper mills that included such occupations as operators, technicians, laboratory workers and repairmen; one was a truck manufacturer that included jobs such as assemblers, mechanics, painters and truck drivers; and one was a steelworks with material handlers, tube workers, repairmen and material preparers. The overall purpose with the AHA-study was to evaluate a comprehensive work intervention concerning its effect on employees’ health and the companies’ productivity. The study was approved by the Ethical Committee of the Karolinska Institute (AHA; Dnr 00-012). Written informed consent of each of the employees was obtained.

During 2000 and 2001 a comprehensive survey was sent to 4 160 employees (3 679 men and 481 women), of whom 2 894 or 70 % (2 523 men and 371 women) responded. The response rates were 77 % among women and 69 % among men, 66 % for blue-collar workers and 89 % for white-collar workers. Among the respondents, 218 employees visited the OHS due to NP/LBP pain during 2000 and 2001. Due to missing values in 18 ÖMPSQ questionnaires, the total score was not calculated and these individuals were excluded. Furthermore, five individuals were excluded since they had been granted part-time disability pension before the time of the OHS examination. The 23 employees that were excluded had a mean age of 40.7 years (SD = 10.2) and 20 (87 %) were men.

### Study Population

The study population consisted of 195 employees. As visible from Table 1, similar to the source population the study group was dominated by males and blue-collar occupations. A vast majority had experienced NP/LBP for more than a year but despite this long-standing pain one-half of the study group only had a week or less of sickness absenteeism. More than a third of the population reported job strain, i.e., high work demands and low job control [25], and slightly more than a quarter of the subjects reported heavy lifting more than 10 times per day.

**Table 1:** Descriptive information on the study population (n = 195)

Characteristics	
Age, mean (SD)	43.1 (9.4)
Gender, n (%)	
Men	162 (83)
Women	33 (17)
Education, n (%)	
Compulsory school	87 (45)
Secondary school	99 (51)
University education	8 (4)
Employment grade, n (%)	
Blue-collar	180 (92)
White-collar	15 (8)
Pain localization, n (%)	
Neck pain	16 (8)
Back pain	34 (17)
Both neck and back pain	145 (74)
Pain duration, n (%)	
≤11 weeks	10 (5)
12–52 weeks	15 (8)
>52 weeks	170 (87)
Pain intensity during last week (1–10), m (SD)	5.4 (2.4)
Sickness absence due to neck-/back pain during the previous year, number of days, n (%)	
0–7	98 (50)
8–30	48 (25)
31–	49 (25)
General health, n (%)	
Excellent/very good	32 (17)
Good	80 (41)
Fair/poor	82 (42)
Job strain, n (%)	73 (38 %)
Perceived physical exertion at work	
Relatively light	37 (19)
Somewhat strenuous	79 (41)
Strenuous or very strenuous	75 (39)
Heavy lifting, n (%)	
Almost never	71 (37)
1–5 times per day	57 (30)
6–10 times per day	10 (5)
More than 10 times per day	55 (28)

### Procedure

Basic demographic information and measurements of workplace characteristics were gathered in the survey that was sent by post to all employees. The 2-year follow-up was also sent by post to employees’ private addresses. However, at one company they also offered the employees the opportunity to complete the survey during working hours. The ÖMPSQ was administered at the OHS at the time for the medical examination. Since the clinical assessment at the OHS was carried out consecutively the exact follow-up period for each employee varied. For the self-reported data the median follow-up time was 25 months (interval 17–32 months). For register data (sickness absence and disability pension) the follow-up time was 24 months for all employees.

At the OHS the employees underwent a medical examination focused on indications of specific diseases, so-called “red flags” [e.g., 26]. The examined employees were advised to stay active and were given a “back book” that offered evidence-based advice on coping with back pain and leading a normal life. Patients with an ongoing sickness absence ≥2 months due to NP/LBP who had not been receiving any rehabilitation during this period were offered 4 weeks of vocational rehabilitation. Patients with recurrent NP/LBP and sickness absence on at least one occasion in the past year, or with an ongoing sickness absence <2 months due to NP/LBP, were offered 2 weeks of vocational rehabilitation. The rehabilitation offered was an in-patient evidence-based multidisciplinary rehabilitation programme (MDP) that has been evaluated among other NP/LBP patients in an earlier study [27]. Seventy-four employees were offered rehabilitation, of which 21 declined. The clinicians had access to the results of the ÖMPSQ but they were not involved in the gathering of the follow-up data in the present study.

### Measurements

The Örebro Musculoskeletal Pain Screening Questionnaire (ÖMPSQ) was developed as a tool to identify individuals at risk for long-term pain and disability among patients with acute or subacute NP/LBP [4]. It comprises 21 items covering such areas as the pain experience, psychological and physical functioning and fear-avoidance beliefs. Most of the items are gathered from other validated questionnaires. Examples of items are “How would you rate the pain you have had during the past week?” (pain experience), “I can do ordinary household chores” (physical functioning) or “An increase in pain is an indication that I should stop what I am doing until the pain decreases.” (fear avoidance). Five items relate to work: sick leave, fear of re-injury due to work, expectations about future work ability, one item about heavy/monotonous work and one item about job satisfaction. The response format for most items is from 0 to 10. The maximum score of each item is ten points and the total score range from 4 to 210 points. Most of the items in the ÖMPSQ were selected from other validated questionnaires since they were considered as potential risk factors for future pain and disability.

The test–retest reliability for the total score of the Swedish version has been reported to be 0.83 [4] or 0.80 [7]. To the authors knowledge the Internal consistency for the Swedish version has not been reported, however, for a Norwegian version of the ÖMPSQ a Cronbach′s alpha of 0.95 was found [8]. Some earlier studies on the ÖMPSQ have suggested cut off scores of 90 [7, 8]or 105 points [4] to differentiate between back pain sufferers with lower or higher risk of future pain, disability or sickness absenteeism. However, findings on the most accurate cut-off scores have varied substantially between studies, probably due to different study groups, contexts and outcomes. For instance, in a recent systematic review on the ÖMPSQ cut off scores from 80 up to 147 points were evaluated [6].

Job strain, co-worker support, support from the superior and monotonous work were assessed with the General Nordic Questionnaire for Psychological and Social Factors at Work, QPSNordic [28]. The internal consistency for the indexes used in this study has been in the range 0.73–0.84 and the test–retest reliability has been in the range 0.78–0.82. The indexes have been shown to be valid [28]. Job strain was constructed based on a median split of the variables job demands and job control, thus employees reporting above the median on both these variables were defined as perceiving job strain. Co-worker support and support from the superior are indexes with an interval from 1 to 5, where 1 denotes that support is very seldom or never at hand and 5 that support is always or almost always given. Monotonous work is a single item including seldom or never monotonous work (1) to always or almost always monotonous work (5). The physical load at work was assessed by single items pertaining to the frequency of heavy lifting, working with hands above the shoulders, proportion of the day exposed to whole-body vibrations, proportion of the day working with handheld vibrating tools [29] and perceived exertion at work [30].

#### Outcome Variables

Registered sick leave: Data on sick leave for the employees were obtained from the companies’ pay registers for the years 2000 to 2003. The data were dichotomized as follows: (1) if the accumulated sick leave was 30 days or more and (0) if the total sick leave was less than 30 days. Thirty days of sick leave has been considered as representing long-term sick leave in earlier research on the ÖMPSQ [4, 7, 8]. Data on disability pension were gathered from the National Social Insurance Board (NSIB) for these same years.

Self-reported sick leave: Current, or previous, sick leave at the 2-year follow up were defined to be the case if the employee responded “yes” to at least one of the items: “Are you currently sick-listed due to neck-/or -back pain?” or “Have you had at least one episode of sickness absenteeism due to neck-/back pain during the previous year?”

Sickness presenteeism was measured according to the following two questions: “Has it happened over the previous 12 months that you have gone to work despite feeling that you should have taken sick leave due to your state of health?” The response format was (1) “No, never”, (2) “Yes, once”, (3) “Yes, 2–5 times”, (4) “Yes, more than five times”. This question has been used in earlier research on sickness presenteeism [16]. Those employees that answered “yes” to this question were also requested to tick in “yes/no” boxes on different health related reasons for their sickness presenteeism. The outcome in this study was coded as (1) if two or more times of sickness presenteeism was reported and no other reasons except NP/LBP were declared for this presenteeism. In all other cases, the outcome was coded as (0).

### Statistical Analyses

The statistical software used in this study was PASW/SPSS 18.0. The ability of the total score of the ÖMPSQ to predict the outcomes was calculated by use of receiver operating characteristic (ROC) curves [31]. The area under the curve (AUC) reflects the ability of the test to discriminate between those with or without a pre-defined outcome or characteristic (e.g., long-term sick leave versus sick leave of shorter duration). A result of 0.50 means no discriminatory ability and a result of 1.0 means perfect discriminative ability. As a guide for interpretation the following criteria were applied: less accurate (0.5–0.7), moderately accurate (0.7–0.9) and highly accurate (>0.9) [32]. ROC curves also were applied to calculate the sensitivity and the “false positive rate” (1-specificity) of the total score of the ÖMPSQ. Additionally, ROC analyses can also be used to see how the sensitivity and specificity alters using different cut off scores. To get some mathematical/statistical guidance in the appraisal of different cut off scores we used positive and negative likelihoods ratios. A positive likelihood ratio describes the odds of arriving at a positive test result in a person with the “condition” in question compared to getting this result in a person without the condition. It is calculated as sensitivity/(1-specificity). A negative likelihood ratio describes the odds of getting a negative test result in a person with the “condition” in question compared to getting this result in a person without the condition. In the interpretations of the LRs, here, as in an earlier study on the ÖMPSQ [8], we give the primary weight to the negative LR, that is, to find a cut off score where the odds of getting “false negatives” are minimised.

To analyse whether workplace factors may alter the predictive strength of the ÖMPSQ, relative risks (RRs) with 95 % CIs for long-term sick leave were estimated by using a modified Poisson regression [33]. To limit the number of analyses, sick leave during the 6 months following the baseline were chosen as the outcome in the analyses of the workplace factors. Sick leave of less than 30 days constituted the reference category. One by one, the workplace factors were tested as the only predictor of the outcome and one by one together with the accumulated score of the ÖMPSQ. Factors that obtained *p* values <0.20, where kept in the analyses. We also tried combinations of different factors. Age and gender were considered as a priori confounders.

### Attrition

#### Sickness Absence

As described, information on sickness absence was available for all employees except those who finished their employment. Twenty-two subjects stopped their employment during the entire 2-year follow-up period. Seventy-three percent of those who stopped their employment were men, the mean age was 49 years (SD = 10) and the mean score on the ÖMPSQ at baseline was 109 (SD = 40) whereas 84 % of those who kept their employment were men, the mean age was 42 years (SD = 9) and the mean score on the ÖMPSQ was 99 (SD = 28).

#### Follow-up Questionnaire

One hundred and forty-four (74 %) individuals responded to the follow-up questionnaire. The mean age among the non-respondents was 44 years (SD = 11), 78 % were men and the mean score on the ÖMPSQ was 109 at the baseline (SD = 36). The 144 respondents had a mean age of 43 (SD = 9), 85 % were men and the baseline ÖMPSQ score was 96 (SD = 26).

## Results

The internal consistency of the total score of the ÖMPSQ was 0.87 (Cronbach′s alpha). The mean ÖMPSQ score at the baseline for the total study group was 100 (SD = 29; range 18–195). For the first 6 months, following the baseline employees with less than 30 days of sick leave had a mean score of 93 (SD = 24) on the ÖMPSQ, whereas those with 30 or more sick leave days scored 129 (SD = 35) on average. For the second year of follow-up the group with 30 or fewer sick leave days also scored 93 (SD = 23) at the baseline and the group with long-term sickness absence scored 112 (SD = 33). *T* tests for independent samples were significant at *p* < 0.001 when the mean scores at the baseline were compared for the different follow-up periods.

### Cut Off Scores

In Table 2 the accuracy of different cut off scores on the ÖMPSQ related to long-term sick leave (≥30 days) is given. Specificity denotes the ability of the total ÖMPSQ score to detect those individuals with a good prognosis and, as visible, it increases with higher cut off scores. Sensitivity, the ability of the instrument to detect those with a poor prognosis with regard to long-term sick leave, decreases with higher cut off scores. For the first 6 months, the best cut off point appears to be 90 points if the purpose is to minimise false negatives (the lowest negative LR). However, if specificity is of high priority, a cut off of 105 may be chosen without losing too much sensitivity.

**Table 2:** Accuracy of the total score of the ÖMPSQ to predict long-term sick leave during the follow-up period

	0–30 Days (specificity)	>30 Days (sensitivity)	−LR^a^	+LR^a^
0–6 months (n = 188)				
90	0.46	0.89	0.24	1.65
100	0.65	0.83	0.26	2.37
105	0.69	0.80	0.29	2.58
110	0.79	0.74	0.33	3.52
0–12 months (n = 184)				
90	0.49	0.85	0.31	1.67
100	0.67	0.71	0.43	2.15
105	0.72	0.69	0.43	2.46
110	0.81	0.60	0.49	3.16
13–24 months (n = 173)				
90	0.48	0.78	0.46	1.50
100	0.65	0.63	0.57	1.80
105	0.72	0.63	0.51	2.25
110	0.81	0.53	0.53	2.78

Examples of potential cut-off scores

^a^ *LR* likelihood ratio

Sensitivity appears to decrease with a longer follow-up period, whereas the specificity remains relatively similar over time (Table 2). For instance, the sensitivity of a cut off score of 90 points decreases from 0.89 for the first 6 months of follow-up to 0.78 for the second year of follow-up. Even for longer follow-up periods a score of 90 renders the lowest negative LR.

Eleven individuals received full-time disability pension during the 2-year follow-up period (four men, seven women). A useful cut off point to detect these individuals was judged to be 130 (Table S1) with the smallest negative LR and a high positive LR. This cut off score has both high sensitivity and high specificity.

At the 2 year follow-up 60 (42 %) of the respondents reported that they were currently on sick leave due to NP/LBP, or that they had been on sick leave during the previous year. The best cut off was as low as 80 points on the ÖMPSQ (Table S2). For sickness presenteeism due to NP/LBP 45 (31 %) employees reported two or more occasions of sickness presenteeism during the previous year and the best cut off score was a score of 95 on the ÖMPSQ (Table S2).

### Predictive Ability

The predictive ability of the ÖMPSQ is described in Table 3. The AUC;s varied from 0.67 to 0.93, which is from less accurate for sickness presenteeism and registered sick leave during the second year of follow-up, to highly accurate for the prediction of disability pension. The ability to predict registered sick leave during the first year of follow up was moderately accurate.

**Table 3:** Area under the ROC curve for sickness absenteeism, disability pension and sickness presenteeism

	Area under the curve	CI (95 %)
Registered long-term sickness absence (≥30 days)		
0–6 months follow-up (n = 188)	0.81	0.71; 0.91***
0–12 months (n = 184)	0.75	0.67;0.84***
13–24 months (n = 173)	0.69	0.59; 0.78***
Full time disability pension (n = 195)^a^	0.93	0.88; 0.99***
Self-reported sickness absenteeism due to neck-/back pain (n = 144)^a^	0.77	0.69; 0.84***
Sickness presenteeism due to neck-/back pain ≥2 occasions during the previous year (n = 144)^a^	0.67	0.58; 0.76***

^a^Two year follow-up

### Workplace Factors

Support from co-workers and support from the superior were the only two workplace factors that had a significant impact on the outcome of long-term sick leave during the 6 months following the baseline. All other workplace factors had *p* values >0.2, regardless of how they were combined with other factors or analysed alone. Due to a strong positive correlation between these two support variables (0.61; *p* < 0.001, Pearson’s correlation coefficient) support from co-workers was excluded from further analyses because it had, compared to support from the superior, a weaker univariate impact on the outcome.

In Table 4, risk ratios for two possible cut offs for the total score of the ÖMPSQ is presented. As can be seen the unadjusted relative risks (RRs) indicate slightly more than a five-fold increase in risk for those scoring above 90 points and a six-fold increase in risk if 105 points is applied as a cut off. Adjusting for gender and age gives a decrease in the RRs of 16–18 %, depending on the choice of cut off and adjusting for support from the superior brings a further slight decrease in RR. However, the score of the ÖMPSQ remains highly significant and indicates a four-fold risk of having long term sick leave during the follow-up period when 90 points is used as a cut off. Consequently, the predictive ability of the ÖMPSQ remains even after adjusting for gender, age and workplace factors.

**Table 4:** Relative risks (RRs) for having registered long-term sick leave (≥ 30 days) during six months following baseline using two different cut off scores of the ÖMPSQ (n = 188)

	n (cases)	RR Unadjusted	RR Adjusted for gender and age	RR Adjusted for gender, age and workplace factors^a^
Sick leave 0–6 months after baseline ÖMPSQ total score				
Cut off 90 points				
≤90	75 (4)	1.0	1.0	1.0
>90	113 (31)	5.1 (1.9; 14.0)**	4.2 (1.5; 11.6)**	4.1 (1.5; 11.3)**
Gender				
Men	156 (22)	–	1.0	1.0
Women	32 (13)	–	1.7 (0.9; 3.1)	1.4 (0.8; 2.7)
Age	188 (35)	–	1.0 (1.0; 1.1)	1.0 (1.0; 1.1)
Support from superior^b^	188 (35)		–	1.5 (1.1; 2.1)**
Cut off 105 points				
≤105	113 (7)	1	1	1.0
>105	75 (28)	6.0 (2.8; 13.1)***	5.1 (2.3; 11.1)***	4.6 (2.0; 10.6)***
Gender				
Men	156 (22)	–	1.0	1.0
Women	32 (13)	–	1.7 (0.9; 2.9)	1.4 (0.8; 2.5)
Age	188 (35)	–	1.0 (1.0; 1.1)	1.0 (1.0; 1.1)
Support from superior^b^	188 (35)		–	1.4 (1.0; 2.0)*

^a^Of the potential confounders only one (support from the superior) fulfilled the criteria to be included in the model

^b^RR; s above 1 indicates higher risk of long-term sick leave when support increases

*** *p* < 0.001; ** *p* < 0.01; * *p* < 0.05

The analyses also showed that more support from the superior was related to an increased risk of long-term sick leave during the 6 months following the baseline (Table 4). Furthermore, rehabilitation was entered as an independent variable together with the total score from the ÖMPSQ; however, this only led to a minor decrease in the RR for the ÖMPSQ. A cut off of 90 points, adjusting for rehabilitation, gave an RR of 4.7 (95 % CI 1.6; 12.0), and for a 105 points cut off the corresponding result was 5.4 (2.5; 11.8).

Finally, since work limitation and sick leave was the focus of this study we also analysed the single item in the ÖMPSQ concerning the amount of sick leave during the year prior to the baseline, dichotomized as 30 days or less (0) versus >30 days (1). The result showed that having more than 30 days of sick leave during the year prior to the baseline increased the risk 11.7 times (CI 5.5; 25.0) for long-term sickness absence 6 months following the baseline.

## Discussion

This study aimed at evaluating the predictive ability of the ÖMPSQ on sickness absenteeism, disability pension and sickness presenteeism among employees examined at the OHS due to NP/LBP. Based on results from ROC analyses and using registered sickness absenteeism as the outcome, the accuracy of the ÖMPSQ remained statistically significant both for the first 6 months, the first year and the second year of the 2-year follow-up period. However, the AUCs indicated that the predictive ability decreased with time. The area under the curve (AUC) varied from 0.67 to 0.93, which is from less accurate for sickness presenteeism to highly accurate for the prediction of disability pension. For registered sick leave during the 6 months following the baseline the AUC from the ROC analyses was moderately accurate (0.81) and a cut off score of 90 renders a high sensitivity but a low specificity whereas a cut off score of 105 improves the specificity substantially but at the cost of some sensitivity. Individuals scoring above 90 points in the ÖMPSQ had a five-fold increase in risk of being on long-term (≥30 days) sickness absenteeism during the 6 months following the baseline. Several workplace factors beyond those included in the ÖMPSQ were considered but only social support at the workplace was significantly related to future long-term sick leave besides the total score of the ÖMPSQ.

If the intention is to minimise false negative results, using the outcome registered sick leave, a cut off score of 90 was the most accurate in this material. This score is lower than found in some other studies on the ÖMPSQ [4, 34]. A score of 90 points has, however, also been suggested in earlier research [7, 8]. A cut off score of 105 may be advantageous if specificity is of higher importance. Differences in “optimal” cut offs may be due to characteristics of the study groups, type of outcome (e.g., sick leave, pain intensity or disability) and different methods of assessing the best cut off. When the intention is to minimise false negatives, this also means that the number of false positives rises. However, as long as the measures for individuals found to be at risk are not too expensive, non-invasive and of no or minimal risk for the patient it appears to be reasonable to accept a number of false positives.

As can be seen in the introduction the CIs around the pooled estimates of the sensitivity and specificity of the ÖMPSQ are wide [6]. Furthermore, in the referred systematic review the heterogeneity for both sensitivity and specificity were high which led the review authors to not recommend the use of one specific cut-off point but instead to build prediction models based on the individual items in the ÖMPSQ. There may also be other alternatives such as using sub scores of the ÖMPSQ for treatment planning and/or for predictive purposes [35] which is further discussed in the systematic review by Sattelmayer et al. [6].

To our knowledge, this is the first study where the predictive ability of the ÖMPSQ has been evaluated concerning future disability pension. The results showed that a highly accurate cut off score for prediction of this outcome was 130 points. Since the ÖMPSQ was developed as a tool in the early prevention of NP/LBP such an outcome as disability pension may be considered as less meaningful. On the other hand, since disability pension is very costly to society, and usually means that the individual will not return to any gainful employment [36], a preliminary knowledge of individuals at risk for this outcome could be used to direct extra resources towards this group.

As a further measure of perceived work limitations, we used future sickness presenteeism due to NP/LBP and the results showed that the ÖMPSQ was also predictive of this outcome. Sickness presenteeism is important since poor health does not necessarily mean sickness absenteeism and an employee’s decision to report sick, or work despite illness, is affected by several factors, both of private, work-related and societal origin [17, 18]. Further, sickness presenteeism may be an overlooked global health assessment in working life, capturing both the individual’s perceived health and his/her working capacity versus the current work situation. In this study, individuals reporting sickness presenteeism due to NP/LBP experienced that they should have been on sick leave but, for some reason, they went to work. From one perspective this may be seen as conforming to evidence-based recommendations to continue everyday activities despite the pain, but the limited evidence that exists concerning actual consequences of sickness presenteeism points in the other direction, i.e., sickness presenteeism has been shown to increase the risk of future sickness absenteeism and poor health [37–39]. Consequently, sickness presenteeism due to NP/LBP may indicate an increased risk of future work limitations and poor general health in the individual.

Analyses were carried out to find out whether the risk of long-term sickness absenteeism predicted by the ÖMPSQ was affected by some potential risk factors in the work environment for sick leave in low back pain patients [22, 23]. However, in univariate analyses of these factors only co-worker support and support from the superior had a significant impact on long-term sick leave. Since these variables were also strongly positively correlated only support from the superior could be used in the final model. After adjusting for age, gender and support from the superior a decrease of 20–23 % in the risk of being on long-term sick leave predicted by the ÖMPSQ could be seen. These results show that the predictive ability of the ÖMPSQ is only affected to a smaller degree by workplace factors, gender and age.

The relation between social support and long-term sickness absence was in the opposite direction to that shown in earlier research [for an overview see 40], i.e., more support was related to higher risk of long-term sick leave. Because of the focus on occupational health in the AHA-project [24] it is possible that both co-workers and supervisors were extra supportive and attentive to those with such health problems that were highlighted in this project, including a heightened acceptance of the use of sick leave as a coping strategy. As reported elsewhere, both superiors and the work staff were involved in, among other things, structured group meetings concerning health and the work environment during the project [24]. Furthermore, there are findings from other research that more emotional/social support has been predictive of more future sickness absence [41]. Consequently, the finding that more social support was associated with an increased risk of long-term sick leave needs replication in other studies before any further conclusions can be drawn.

Concerning the generalizability of results, a few points need to be considered. The majority of employees in the study group were reporting a pain duration of more than 52 weeks and both NP and LBP. Additionally, all participants reported NP or LBP already at the time of the foregoing survey (see Methods). Based on this, many individuals in the study group appear to suffer from chronic pain. However, half of the study population reported 7 days or less of sickness absenteeism during the year prior to the baseline, and a further quarter of the study group describe less than 30 days of sick leave, thus the work ability appears to be good or relatively good for the majority of the participants. The descriptive data on pain duration and sickness absenteeism may indicate that the participants were at different stages in their pain course. This could introduce some uncertainty about the interpretation of the ÖMPSQ since different cut off values may be needed for different patient groups based on earlier pain duration or sickness absenteeism. Nevertheless, these characteristics may be applicable for individuals seeking help for NP/LBP at the OHS within the industrial sector. It could also be added that, since prevention of NP/LBP was one of the focus areas of the AHA-study (see Methods), some employees who should not normally have visited the OHS did so because of the study. Therefore, with some caution, the results should be generalizable to similar male-dominated industries.

The results of this study extend and confirm the findings of earlier research on the ÖMPSQ. Routine assessment of psychosocial risk factors among employees seeking help for NP/LBP at the OHS could be helpful in finding those at risk for future work disabling problems. The ÖMPSQ may be used in a “stepped care approach” where workplace visits and further clinical interviews are also included [40]. Effective measures for the prevention of work limitations among NP/LBP represent the next step or challenge in this process [42, 43].

## Electronic supplementary material

Below is the link to the electronic supplementary material.

Supplementary material 1 (PDF 93 kb)
