# Supplementary material for: Prediction of Sickness Absenteeism, Disability Pension and Sickness Presenteeism Among Employees with Back Pain
Source: J Occup Rehabil. 2013 Jun 18;24(2):278–86. doi: 10.1007/s10926-013-9454-9 (PMC4000420; doi:10.1007/s10926-013-9454-9)
Supplement: Supplementary file 1 — Supplementary material 1 (PDF 93 kb) [file 10926_2013_9454_MOESM1_ESM.pdf]

Online Supplementary Tables

Online Table 1. Accuracy of the total score of the ÖMPSQ to predict disability pension during the follow-up period. Examples of potential cut-off scores.

	No disability pension (specificity)	Granted disability pension (sensitivity)	-LR	+LR
24 month follow-up				
105	0.64	1.0	-	2.77
110	0.72	0.91	0.12	3.25
130	0.89	0.91	0.10	8.27
140	0.94	0.73	0.29	12.16

Online Table 2. Accuracy of the total score of the ÖMPSQ to predict self-reported sickness absenteeism and sickness presenteeism at the two-year follow-up.

	No sickness absenteeism (specificity)	Sickness absenteeism (sensitivity)	-LR	+LR
Self-reported sickness absenteeism[a]				
80	0.44	0.95	0.11	1.70
85	0.48	0.87	0.27	1.67
90	0.56	0.80	0.35	1.82
105	0.79	0.57	0.54	2.71
	No sickness presenteeism (specificity)	Sickness presenteeism (sensitivity)	-LR	+LR
Sickness presenteeism[b]				
90	0.48	0.73	0.56	1.40
95	0.60	0.71	0.48	1.78
100	0.68	0.58	0.62	1.81
105	0.71	0.49	0.72	1.69

[a] = Currently sick-listed due to neck-/or back pain and/or having had at least one episode of sickness absenteeism due to neck-/back pain during the previous year.

[b] = Sickness presenteeism due to neck-/back pain on at least two occasions during the previous year.
